# Electromagnetic Characterization of EAC-1A and JSC-2A Lunar Regolith Simulants

**DOI:** 10.3390/ma17153633

**Published:** 2024-07-23

**Authors:** David Ramos Somolinos, Borja Plaza Gallardo, José Cidrás Estévez, Narek Stepanyan, Aidan Cowley, Alicia Auñón Marugán, David Poyatos Martínez

**Affiliations:** 1 Radiofrequency Area, National Institute for Aerospace Technology (INTA), 28850 Madrid, Spain; dramsom@inta.es (D.R.S.); plazagb@inta.es (B.P.G.); aaunmar@inta.es (A.A.M.); 2 School of Engineering, Universityof Alcalá, UAH, 28801 Madrid, Spain; jose.cidras@edu.uah.es (J.C.E.); narek.stepanyan@edu.uah.es (N.S.); 3 European Astronaut Centre, EAC, Linder Höhe, D-51147 Cologne, Germany; aidan.cowley@esa.int

**Keywords:** regolith, EAC-1A, JSC-2A, electromagnetic characterization, permittivity, permeability, tangent loss

## Abstract

The development of devices for the in situ resource utilization (ISRU) of lunar surface powder (regolith) by means of microwaves needs regolith simulants with electromagnetic properties similar to the lunar regolith. This document deals with the measurement of complex permittivity and dielectric loss tangent of the aforementioned simulants at ambient temperature from 400 MHz to 20 GHz, performing measurements using two lunar dust simulants, EAC-1A and JSC-2A, resulting, on the one hand, in permittivity values of $\varepsilon^{\prime }=-0.0432f+4.0397$ for the EAC-1A lunar dust simulant and $\varepsilon^{\prime }=-0.0432f+4.0397$ for the JSC-2A simulant, and on the other hand, in loss tangent values of $tan\delta_{e}=-0.0015f+0.0659$ for the EAC-1A powder and $tan\delta_{e}=-0.0039f+0.1429$ for the JSC-2A powder. In addition, further studies are carried out taking into account the humidity of the samples and their densities at room temperature. The obtained results are applicable for comparing the measured values of EAC-1A and JSC-2A between them and with other previously measured simulants and real samples. The measurements are carried out by applying two different nonresonant techniques: Open-Ended Coaxial Probe (OECP) and transmission line. For this purpose, the DAK and EpsiMu commercial kits are used, respectively.

## 1. Introduction

One of the reasons why it is important to characterize electromagnetically lunar regolith (lunar surface dust) is to be able to use this material as an in situ resource (ISR) [1,2,3]. This can mean a turning point for the colonization of territories outside our planet, allowing the human race to gain knowledge about new materials which could lead into a more frequent presence on the Moon and enable new possibilities in terms of exploration of the Universe [4].

In response to this, several uses of regolith or lunar dust can be highlighted such as the possibility to obtain oxygen [5] and water [6,7] for habitability, as well as the use of water for protection against radiation for missions that extend in time outside the protection of the earth. Also, hydrogen [8] and oxygen can be extracted from it for space propulsion and electricity generation [9].

Moreover, lunar regolith can also be employed as a building material [10] once sintered, which is of great interest in this study.

To sinter regolith, it is necessary to heat it up, and an effective way to achieve this is through radiation at microwave wavelengths [11,12]. Therefore, in order to design a functional device for microwave frequencies to process the regolith, it is mandatory to carry out a previous analysis to figure out the frequency at which the electromagnetic energy transferred to the dust is more easily converted into heat. The characterization of regolith is based on knowing electromagnetic properties such as permittivity, permeability and loss tangent, at the widest frequency range possible. Studying the loss tangent and finding its maximum values (or how it varies) can provide insight about what the most efficient frequency range is to irradiate the regolith, thus establishing design guidelines for future microwave processing equipment. Moreover, there have not been many missions to the Moon yet, which means that there is a shortage of these materials and hence, performing extensive measurement campaigns testing samples of lunar regolith is not a feasible option. Nevertheless, the solution that NASA and other public and private companies have devised is to simulate these lunar dusts through materials that we have on Earth [13], so that these dusts can be measured, even with destructive tests. These new powders that simulate the lunar regolith are known as simulants and several of them have been developed [14], as well as Martian surface simulants [15], since, as it is known, the next step after settling on the Moon will be the arrival of human beings on the Martian surface. These lunar simulants differ in their composition; this is because the lunar dust is not homogeneous over the entire Moon, so it is mandatory to have different simulants so that the entire surface of the Moon can be characterized.

In addition to characterizing regolith samples, various studies focusing on investigating the dielectric properties of different celestial bodies can be found in the literature. One example is [16], where research is conducted into the dielectric properties of both the nucleus and the materials present on a comet’s surface by means of radar remote sensing, showing measurement methodologies that can be applied to studies carried out on lunar simulants.

**Figure 1:**
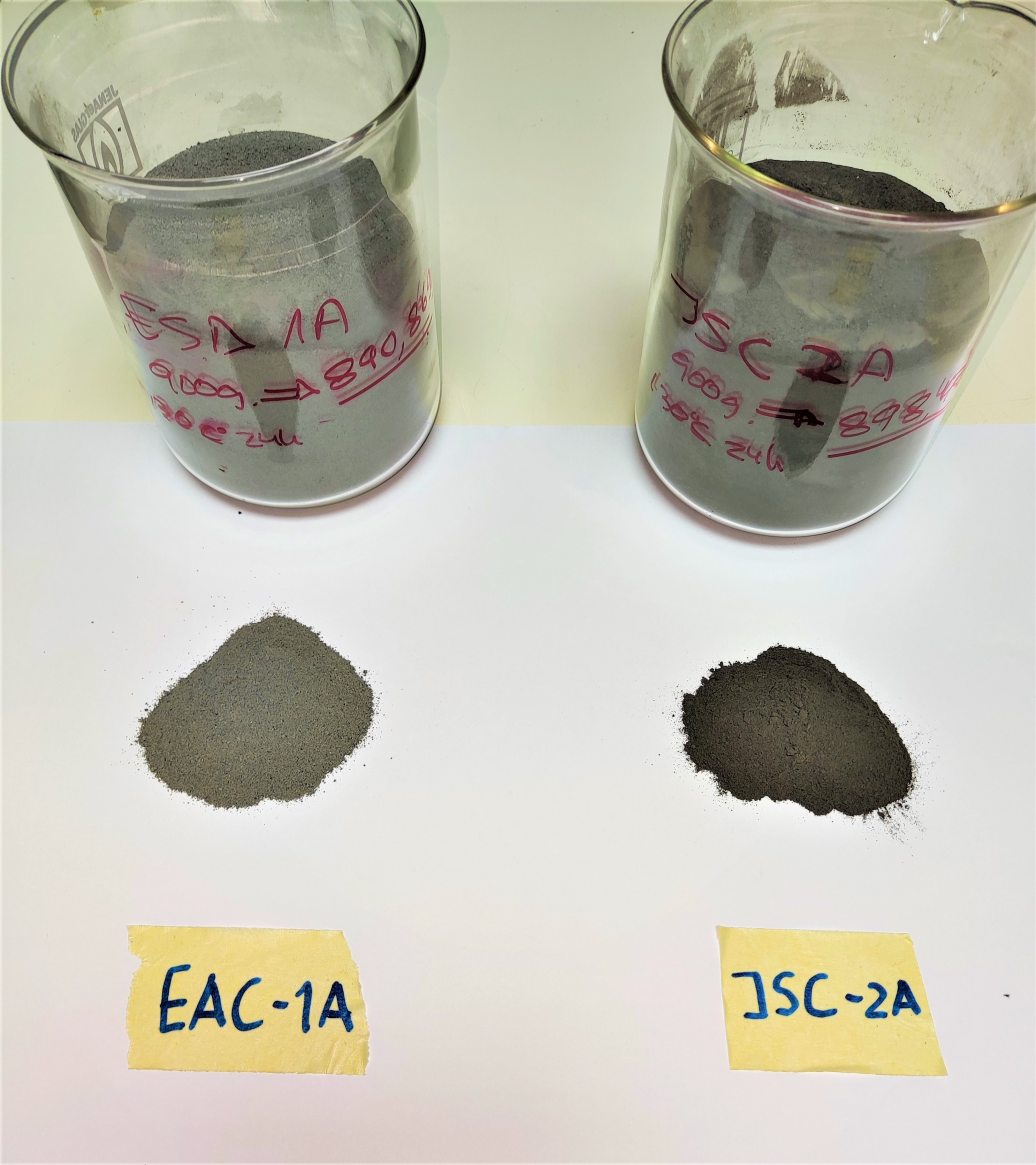
Sample of lunar regolith simulants EAC-1A **(left**) and JSC-2A (**right**).

Under these circumstances, this work is focused on electromagnetically characterizing two lunar dust simulants, namely, EAC-1A [17] (which is one of the first simulants developed by ESA’s European Astronaut Center) and JSC-2A [18] (developed by NASA at Johnson Space Center) (see Figure 1), by means of analyzing their complex permittivity and permeability values. To do so, two different measurement methods, the OECP method (Open-Ended Coaxial Probe) and the coaxial transmission line method are employed. The first test is carried out simply by taking measurements at room temperature. Later, this study is expanded in two ways, one considering variations in compaction for the powders, which reduce the amount of air in the sample, varying its bulk density, and another one consisting in analyzing how drying the simulants may affect the final results of the real part of the relative permittivity and the loss tangent.

There have been previous efforts in this regard. Among them, eight regolith simulants and three sample of actual lunar regolith are selected in this study to provide context and serve as comparative values for the EAC-1A and the JSC-2A powers, which are JLU-1 [19], JSC-1 [20], JSC-1A [21], JSC-1A(1% CIP) [22], JSC-1A (AGGL) [14], LHS [23], LMS [24] and CAS-1 [25] as simulants, and 70051 [26], 14163 [27,28] and CE [29] as lunar regolith samples. As it is explained further in this article, all the different samples, simulants or real, exhibit a dielectric behavior, having values of the real part of the relative permittivity ranging from 2 to 4, and showing low values of loss tangent, ranging from 0 to 0.16.

In addition, ESA is developing a facility called LUNA for prospective lunar missions’ payload and technology development and testing. Its ground has to be filled with a regolith simulant, and EAC-1A is a likely candidate for that propose [17].

## 2. Measurements Methods

Electromagnetically characterizing a material means knowing the interaction of that material with electromagnetic radiation. The way in which this interaction occurs can be known through two parameters, which are the complex relative permittivity, shown in Equation (1), and the complex relative permeability, shown in Equation (2) [14]:

(1) $$\varepsilon_{r}=\varepsilon_{r}^{\prime }-i\varepsilon_{r}^{\prime \prime }$$

(2) $$\mu_{r}=\mu_{r}^{\prime }-i\mu_{r}^{\prime \prime }$$

where $\varepsilon_{r}$ is the relative permittivity, $\varepsilon_{r}^{\prime }$ is its real part, and $\varepsilon_{r}^{\prime \prime }$ is its imaginary part. Similarly, $\mu_{r}$ is the relative permeability, $\mu_{r}^{\prime }$ is its real part, and $\mu_{r}^{\prime \prime }$ is its imaginary part.

Once the parameters that are necessary to characterize a material have been defined, it remains to be determined what method to use to obtain the values of these variables. This choice is made upon the analysis of different criteria such as the frequency of interest, the accuracy needed to make a correct measurement, the temperature, the size of the sample to be tested, the kind of material being studied or whether the test can deteriorate the sample of material or not [30]. Taking into account the material to be characterized (in powder form) and wanting to know its behavior against electromagnetic fields in the widest frequency range, it was decided to use two types of nonresonant measurement techniques [31]. These two methods were the OECP method and the coaxial transmission line method.

As mentioned in the Introduction, in this work, three types of tests were carried out. The first was to find out the electromagnetic properties of both simulants, compared to the other samples mentioned in the Introduction. The second consisted in analyzing the behavior of those electromagnetic properties before and after heating the EAC-1A and JSC-2A. And finally, the third test involved performing measurements for different densities of the powders. It is important to mention that all these experiments were carried out at room temperature as a first approach to validate the measurement methods, but in this regard, further analysis could be taken in the future using the environmental chambers available at the National Institute for Aerospace Technology (INTA) [32].

### OECP and Transmission Line Methods

The OECP method [33,34] is a non-destructive method that measures the reflection parameters of the sample under test. Since it is mainly designed to work with dielectric samples of materials, it was used for the extraction of the permittivity and loss tangent (see Equation (3)).

(3) $$tan\delta_{e}=\frac{\varepsilon^{\prime \prime }}{\varepsilon^{\prime }}$$

where $tan\delta_{e}$ is the loss tangent, which is the ratio between the imaginary part of the permittivity, $\varepsilon^{\prime \prime }$, and its real part, $\varepsilon^{\prime }$.

To carry this test out, the device selected was the commercial measurement kit from the company SPEAG (Zurich, Switzerland) [35], called DAK-3.5 (Dielectric Assessment Kit), which allowed us to obtain results up to 20 GHz and provided us with software developed by the same company to estimate the values of the aforementioned permittivity and loss tangent of the material.

**Figure 2:**
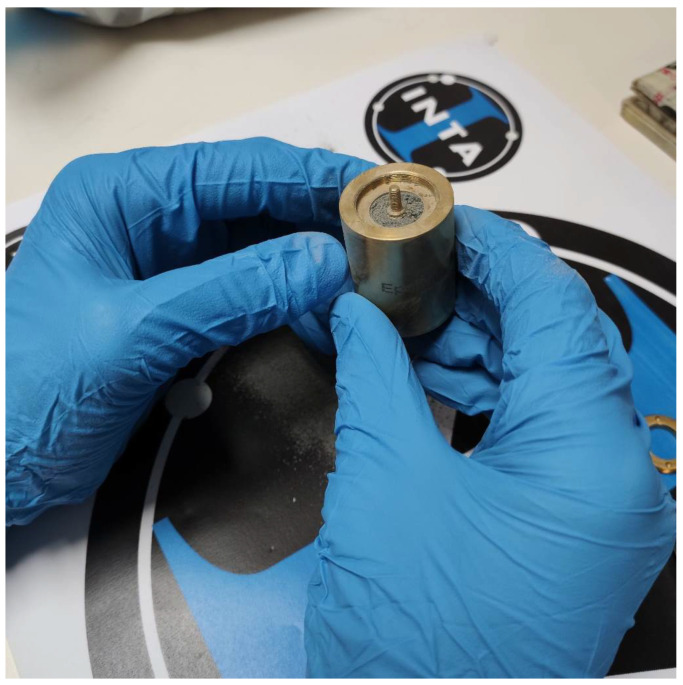
EpsiMu PE 13 mm sample holder with lunar regolith simulant inside.

For the transmission line method, another commercial kit called the EpsiMu 13 mm measurement kit (EpsiMu, Lichtenau, Germany) [36] was used. With this kit, both reflection and transmission parameters can be obtained [37], but in order to do so precisely, the lunar dust must fill the whole sample holder (30.10 mm), as it is shown in Figure 2. Using this method, both permittivity and permeability can be obtained, but the validity of the results can only be claimed up to a certain frequency, which varies according to the electromagnetic properties of the sample. This behavior can be explained with the following expression [36]:

(4) $$\frac{\lambda }{2}=\frac{c}{2f}\frac{1}{\sqrt{\varepsilon \mu }}$$

where λ is the wavelength, *c* is the speed of light in vacuum and ε, and μ are the real parts of the permittivity and permeability of the dust. In this case, for a sample size of 30.10 mm, the highest frequency achievable is 2.5 GHz. On the other hand, if the material to be measured is non-magnetic (μ = 1), this range may be extended to 6 GHz.

A more detailed description of the named kits, the calibration performed, and how the measurements were made are detailed in [38].

## 3. Results and Discussion

The obtained values of permittivity and dielectric loss tangent for the studied JSC-2A and EAC-1A simulants are shown in addition to other lunar regolith and simulants obtained from the literature. Considering that for frequencies below 400 MHz, those values became unstable, all the measurements of the JSC-2A and EAC-1A simulants were carried out at room temperature in a range between 400 MHz and 20 GHz. Before carrying out the measurements, the density of the materials was also estimated, obtaining a density at atmospheric pressure of 1.72 g/cm^3^ for the EAC-1A simulant and a value of 1.73 g/cm^3^ for the JSC-2A simulant.

The results for the magnetic permeability and tangent of magnetic losses have been previously published in [38]; therefore, the graphs corresponding to these values are not shown in this study. From these graphs, it was possible to conclude that the simulants JSC-2A and EAC-1A were non-magnetic, with a permeability of one and a value in the tangent of magnetic losses close to zero.

In addition, in [38], the results of permittivity and tangent of dielectric losses were also shown. In that case, a linear approximation of these results was used. The values obtained can be seen in Table 1 and Table 2. In general, adequate values were obtained according to the two simulants studied, both in the real part of the permittivity and in the loss tangent. A greater variation at low frequencies was observed by means of the DAK-3.5 kit (lower frequencies imply larger wavelengths, and the size of the sample to be measured was not large enough so the electromagnetic field had areas outside the material, and the dielectric properties obtained were not adequate).

### Comparison of Dielectric Properties

The approximation was divided into two parts. On the one hand, in the case of the measurements performed using the DAK-3.5 kit, for the frequency range from 6 GHz to 20 GHz, trend lines were generated. On the other hand, for the EpsiMu measurement kit, those trend lines ranged from 500 MHz to 6 GHz. The error introduced when linearly approximating the measurements was also estimated. For the case of the adjustment from 0 to 6 GHz, there was a maximum dispersion of ±0.1 in permittivity and ±0.015 for the dielectric losses. From 6 to 20 GHz, there was a dispersion of ±0.2 for the permittivity and ±0.07 for the loss tangent.

The results of both regolith simulants (EAC-1A and JSC-2A) were compared with the real part of the relative permittivity and the dielectric loss tangent values of other simulants analyzed in different studies. Additionally, the simulants measured in this study were also compared to actual lunar regolith samples

Before starting the analysis, it is important to mention that both simulants replicate the lunar mare basalt regolith [18,39]. There are other simulants that recreate different parts of the lunar surface, such as the highlands, but at the time of this study, they were not available and that is the reason why the measurements showed in this article were carried out using only the EAC-1A and JSC-2A powders. However, the same measurement methods described in this study could be applied for them, regardless of the composition.

Despite both EAC-1A and JSC-2A being mare regolith samples, in the comparison performed in this work, powders with different compositions were included. This decision was made to find out how much the behavior in terms of permittivity and loss tangent may differ, so a more comprehensive knowledge could be achieved. Again, the tests carried out in this study could be extended if new simulants are available.

First, our simulants were compared with those listed in Table 3. As can be seen, there were a total of five different lunar simulants and a sample of lunar regolith corresponding to the Apollo 17 mission. The simulants in this comparison were, on the one hand, the JLU-1 simulant [19], which is a lunar highland simulant developed at Jilin University, China, and, on the other hand, the JSC-1 simulant and its variants JSC-1A, JSC-1A (1% CIP) and JSC-1A (AGGL) [14,22]. Simulants JSCs were no longer manufactured and was replaced by JSC-2A, as it represented a more advanced simulant, with properties closer to that of real lunar regolith according to [40].

This first comparison was published at the 2022 EuCAP (European Conference on Antennas and Propagation) in Madrid [38], where the properties of each of the named samples were detailed. However, in order to extend this comparison, new simulants and samples of real lunar regolith were added in this study. For this new comparison, three new types of simulants and two other types of lunar regolith were added. The new measured samples can be seen in Table 4.

**Figure 3:**
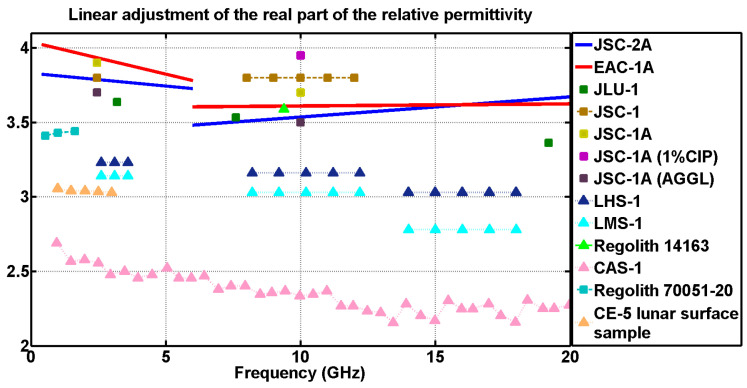
Linear fit of the real part of the relative permittivity of the EAC-1A and JSC-2A simulants (data from 0.5 GHz to 6 GHz obtained from the EpsiMu kit and data from 6 GHz to 20 GHz obtained from the DAK-3.5 kit).

**Figure 4:**
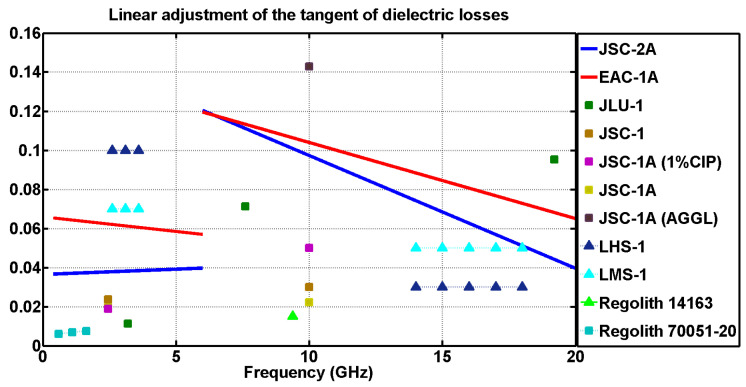
Linear fit of the dielectric loss tangent of the EAC-1A and JSC-2A simulants (data from 0.5 GHz to 6 GHz obtained from the EpsiMu kit and data from 6 GHz to 20 GHz obtained from the DAK-3.5 kit).

In order to differentiate the samples used in this study from those shown in [38], the real permittivity and loss tangent values of the newly added samples are represented by triangles, while the others appear as squares. The results of the set of samples corresponding to Table 3 and Table 4 are presented in Figure 3 and Figure 4.

The first comparison made was based on the LHS-1 [23] and the LMS-1 [24] simulants, both manufactured at the Exolith Lab, which is a not-profit organization largely funded by CLASS, the Center for Lunar and Asteroid Surface Science. The measurements of these two simulants were carried out in three frequency bands, S-band (2.6–3.95 GHz), X-band (8.2–12.4 GHz), and Ku-band (12.4–18 GHz), using the waveguide method [15]. The relative permittivity and dielectric loss tangent values of the LHS-1 and LMS-1 correspond to measurements at room temperature and for different concentration values of the samples. Therefore, in the present study, the values chosen for the comparison were those corresponding to the mean concentration values of the material, which appear in Table 4 of [15].

Another simulant with which the studied powders were compared was CAS-1. This lunar soil simulant was designed by the Chinese Academy of Sciences, to support lunar orbiter soft-landing mission and sample return missions of China’s Lunar Exploration Program [25]. Source material for this simulant was a low-Ti basaltic scoria from the volcanic area in the Changbai Mountains of Northeast China. Consistent with [25], the scoria was analyzed by XRF (X-ray fluorescence) and found to be chemically similar to lunar sample 14,163 from Apollo 14, and at low frequencies (less than 1 GHz), it appeared to have a similar permittivity value. Nevertheless, as can be seen in Figure 3, at 9 GHz the real permittivity value of CAS-1 was well below the value corresponding to the regolith 14,163 sample. It can be seen that the Lunar regolith from Apollo 14, considering the complex permittivity, had a behavior closer to the studied simulants JSC-2A and EAC-1A in the X-band.

Once the simulants provided in the comparison had been described, the lunar regolith samples remained to be defined. First, there was the lunar regolith corresponding to the Apollo 14 mission. The sample of this regolith was 14163-164 and the measurements shown in Figure 3 and Figure 4 correspond to the complex relative permittivity at 9.375 GHz. These values were obtained by the short-circuit wave guide technique, where the sample was compressed until reaching a bulk density of 1.71 g/cm^3^ [41], a value similar to the case of the simulants JSC-2A and EAC-1A analyzed (1.73 g/cm^3^ and 1.72 g/cm^3^, respectively). Considering the latter, it can be seen that at 9.375 GHz, the values of the real part of the relative permittivity of the two regolith simulants, EAC-1A ($\varepsilon^{\prime }$ = 3.61) and JSC-2A ($\varepsilon^{\prime }$ = 3.53), were similar to that of the Apollo 14 regolith sample ($\varepsilon^{\prime }$ = 3.59).

Furthermore, there are also data on the complex permittivity values of sample 14,163 with higher bulk density values. According to [41] with a bulk density of 1.9, the value of the real part of the permittivity becomes 4.1. This increase in permittivity with increasing compaction in the powders is expected, and the variation in this parameter is similar to the variation obtained in this study with the EAC-1A and JSC-2A simulants.

The other type of lunar surface sample was CE-5. This material corresponds to the regolith brought to earth from the third phase (Chang’E-5) of the China Lunar Exploration Program (CLEP). The displayed measures of the real part of the permittivity and tangent of dielectric losses of CE-5 were found in a frequency range from 1 to 3 GHz and were measured at a stable temperature of 26 °C [29]. In addition, the measurements were made with the transmission line technique, and the bulk density value that corresponded to these measurements was 1.58 g/cm^3^, so according to the study carried out in Section 4.2, at that density, the real part permittivity value of 3.04 appears to be in agreement with the simulants JSC-2A and EAC-1A.

Analyzing the results obtained from the measurements performed with the DAK-3.5 and the EpsiMu commercial kits, depicted in Figure 3 and Figure 4, it was proved that the dielectric parameters (permittivity and loss tangent) of EAC-1A and JSC-2A powders are comparable to the those of rest of the simulants, which validates the results.

In the case of the real part of the permittivity (Figure 3), it can be seen that the values were quite close to those of other simulants, where the EAC simulant seemed to have a higher permittivity value than the JSC simulant, and in the case of the loss tangent (Figure 4), the values obtained were slightly above those expected.

For the case of a range from 6 to 20 GHz, it was again observed that in the case of the real part of the permittivity, the values were close to those of the other simulants (Figure 3), and for the loss tangent (Figure 4), a greater dispersion was observed.

The differences found in the measurement of the loss tangent discussed above are a consequence of accuracy issues. This phenomenon can be explained because the loss tangent relates the real part of the permittivity to its imaginary part. Since the measurement methods employed in this work were nonresonant, they provided lower accuracy when obtaining the imaginary part of the permittivity, compared to the resonant methods employed in the literature, so inevitably, there was a larger uncertainty in the loss tangent. Nevertheless, in this research, a wider range of frequencies was analyzed.

When attempting to explain the behavior of the powders, it should be borne in mind that their electromagnetic properties vary widely depending on the composition of the powders. EAC-1A’s composition was studied in [39], giving a result of 43.7% SiO_2_, 11.9% MgO, 4.2% (Na_2_O + K_2_O) and 2.4% TiO_2_, whereas for JSC-2A, it is known that it contains up to 50% SiO_2_ and less than 4.5% Ti [18]. In this way, even though the behavior of both simulants exhibited in Figure 3 and Figure 4 is strongly related to their mineral composition, the authors of this work lack the experience to properly correlate those values of permittivity and loss tangent with respect to the different materials composing the powders, so the results presented in this article are open to discussion and analysis by the scientific community.

Once the permittivity and loss tangent results are known, it must be addressed how these parameters affect the process of microwave sintering. Among the different sintering techniques, the most conventional ones are based on conduction, radiation or convection [42], where the surface of the material is heated, and the energy is transferred inwards. However, with microwave sintering techniques, the opposite happens; the energy is transferred first into the material, and then it travels to the rest of the volume, reducing the energy consumption [43]. If a microwave sintering process is selected, it must be considered that the amount of electric energy the material is capable of storing depends on permittivity. High values of the real part of this parameter imply low values of penetration depth of microwaves, and vice versa [44], so the heating process is more efficient for powders with lower values of the real part of the permittivity. Moreover, while the real part of the permittivity shows the capacity of the material to store the electric energy, the imaginary part indicates its ability to dissipate that energy into heat, both being related through the loss tangent, so higher values of the loss tangent inform at which frequencies the sintering is more effective.

## 4. Other Studies

In order to continue advancing in the characterization of these materials, the study was expanded by adding two new sections, which provide relevant information on their behavior in the face of changes in compaction and in the amount of water content.

On the one hand, variations in compaction imply variations in bulk density and this means obtaining higher permittivity values by reducing the amount of air in the measured sample.

Variations in the amount of water have the opposite effect. Measurements of lower permittivity values are expected, since once the previous measurements have been made, it is known that the water has a much higher permittivity than the simulants (at room temperature, water has a relative permittivity value close to 80).

### 4.1. Drying Lunar Simulants

For the following case, 900 grams of both powders was heated in an industrial oven for 24 h at 130 °C. Once heated, they stood long enough to return to room temperature and were reweighed in order to measure the percentage of water lost.

After heating, 890.70 g of EAC-1A and 898.24 g of JSC-2A remained. This represented a loss of approximately 1% for the EAC-1A simulant (9.3 g) and a loss of 0.2% for the JSC-2A simulant (1.76 g).

**Figure 5:**
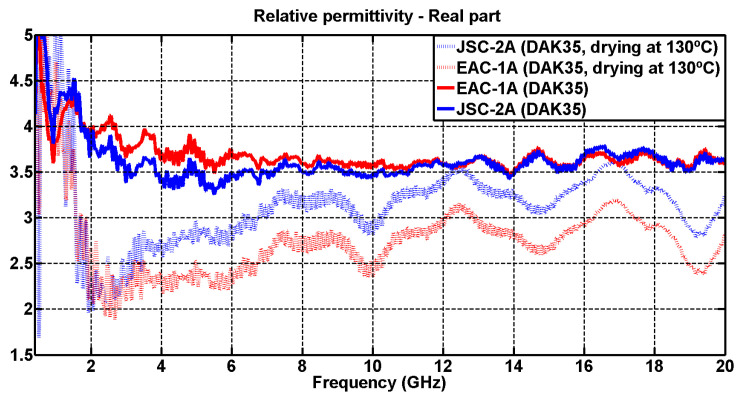
Comparison of the real relative permittivity measurements between the dried and the undried simulants (DAK-3.5 kit).

**Figure 6:**
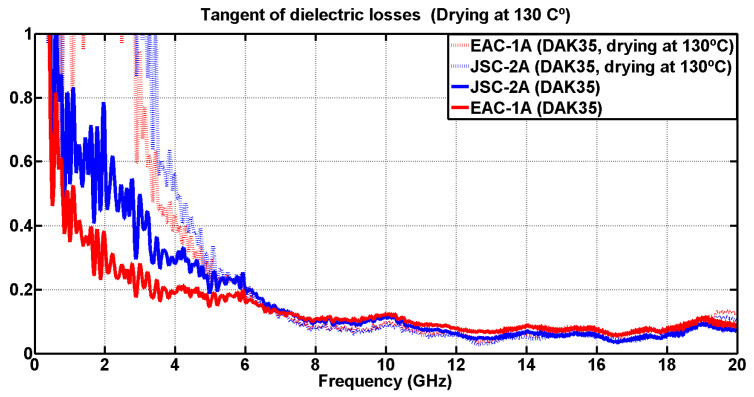
Comparison of the tangent of dielectric loss measurements between the dried and the undried simulants (DAK-3.5 kit).

The variations obtained are displayed in Figure 5 and Figure 6, so that the values of the undried powder can be compared with the values of the dried powder. All measurements in this section were carried out using the DAK-3.5 kit, already discussed throughout this study, in a single day in order to avoid variations in the amount of water lost.

As can be seen in Figure 5, the expected result was obtained. The real part of the relative permittivity decreased for the dried simulants compared to the non-dried simulants.

On the other hand, it can be observed that the simulant with the greatest loss in the percentage of water (EAC-1A) had a greater decrease in the value of the real permittivity (red dashed line), which gives more validity to the hypotheses made on the relationship between the amount of water in the powders and the loss of dielectric permittivity.

Regarding the loss tangent shown in Figure 6, for the lowest frequency range up to 6 GHz, there was a large measurement dispersion. However, at higher frequencies, it can be seen that these measurements stabilized and the variation in results between the dried and non-dried powders was practically non-existent.

### 4.2. Study on Variations in Density

To carry out the measurements of this section, only the EpsiMu kit was used, since it was the only one that allowed a measurement of the different relative densities of the powder, depending on the amount that was incorporated inside the sample holder (Figure 2).

Therefore, for the calculation of the density, different amounts of powder were added in the sample holder to be weighted, so that by obtaining the difference with respect to the weight of the sample holder alone, the weight of simulant that had been introduced could be determined. Later, the density was calculated by dividing those weights by the volume of the sample holder.

**Figure 7:**
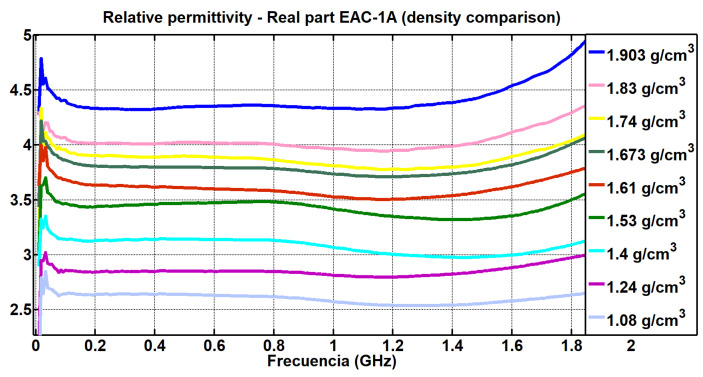
Comparison of the real part of the relative permittivity obtained by varying the density of the simulant EAC-1A (EpsiMu kit).

**Figure 8:**
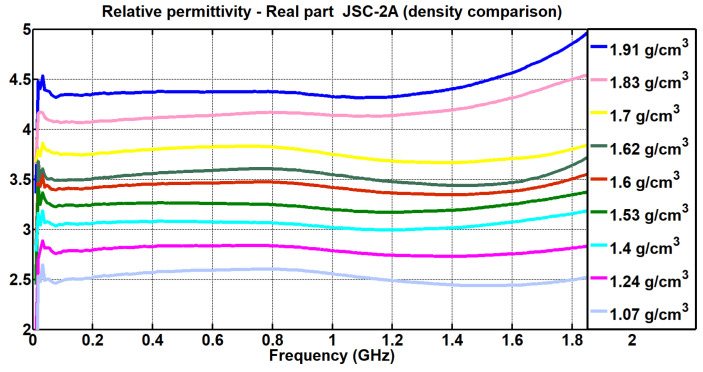
Comparison of the real relative permittivity measurements when varying the density of the simulant JSC-2A (EpsiMu kit).

The values obtained from the real part of the relative permittivity for each density and for each simulant can be seen in Figure 7 and Figure 8.

Figure 7 depicts the behavior of the permittivity of the simulant EAC-1A. It can be seen that as the density decreased, there was a lower value of the real relative permittivity. Likewise, Figure 8 shows the same analysis for the JSC-2A powder. Again, it is observed that lower values of density corresponded to lower values of the real part of the relative permittivity.

In Figure 7 and Figure 8, it is observed that as the density decreased, there was a lower value of the real permittivity. It can be seen that from frequencies close to the cut-off frequency (2.5 GHz) mentioned in Section 2 of this study, the measurements diverged, although throughout the entire range between 400 MHz and 1.6 GHz, the measurements were linear.

**Figure 9:**
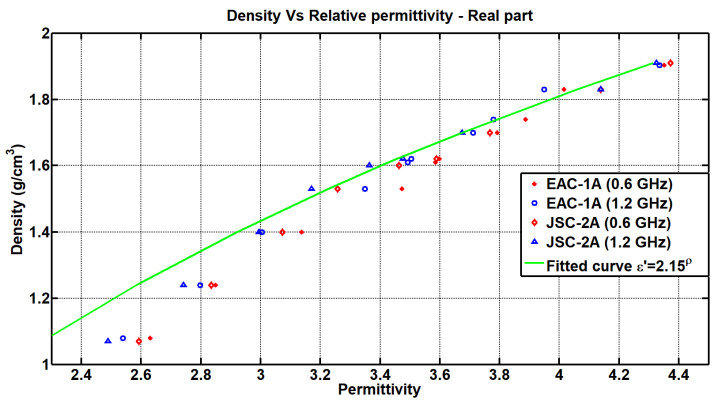
Comparison of the real part of the permittivity measurements of the EAC-1A and JSC-2A regolith simulants when varying the densities (EpsiMu Kit [0.6 and 1.2 GHz]), with respect to the fitting curve from [14].

Finally, the results obtained were compared with those of other studies carried out on the variation in density of the simulants. In [14], a large number of measurements are carried out with different simulant densities, and an approximation curve of these measurements was reached, which related the density variations as a function of the real part of the permittivity as follows:

(5) $$\varepsilon^{\prime }=2.15^{\rho }$$

where ρ is the density. This theoretical equation does not vary with frequency, since, as seen previously, the variation in density (in the case of the real part of the permittivity) is linear with respect to different frequencies. In this way, two frequencies were chosen (0.6 MHz and 1.2 GHz), and the results obtained for the real part of the permittivity and density variation were compared with the approximation extracted from [14] (Figure 9). As can be seen, the data obtained in our measurements were approximated with the aforementioned fitting curve.

## 5. Conclusions

This study tried to address the growing interest from the space industry in knowing the behavior of lunar dust against electromagnetic radiation, since it can be observed worldwide how the number of space programs to return to the moon has grown considerably.

Two types of lunar simulants (EAC-1A and JSC-2A) were electromagnetically characterized and both of them showed a similar mineralogy to other simulants and real regolith samples extracted from the moon. Two independent measurement methods were used (OECP and transmission line), from the DAK and EpsiMu measurement kits, respectively. Using them, it was possible to characterize the JSC-2A and EAC-1A simulants in a frequency range between 400 MHz and 20 GHz. This frequency range is especially wide taking into account the characterization seen for other simulants in the literature. To validate the results obtained from our measurements, a comparative study was carried out. In it, the differences between the permittivity values obtained from our simulants with respect to others present in the literature were seen and, in general, it was possible to conclude that the measurements made were consistent.

Based on these results, it was observed in the case of the real part of the relative permittivity that the simulants studied were similar to the JLU, JSC simulants and their variants, and that the simulant CAS-1 was far from these values.

On the other hand, considering the LHS-1 and LMS-1 simulants, a greater difference in the permittivity value was also observed. However, this difference could be attributed to compaction when measuring the powders. In this sense, it was observed in the data extracted from [15] that with in higher compaction, permittivity values close to those seen in the case of JSC-2A and EAC-1A were reached.

In the case of real lunar regolith samples, it can be concluded that the sample corresponding to Apollo 14 and 17 had a greater resemblance to the simulants studied, while in the case of the Chinese CE-5 regolith, the resemblance was more consistent with the simulants LHS-1 and LMS-1.

Among the multiple research lines that can be followed to obtain a better insight into the behavior of the powders, controlling the temperature and the percentage of water inside them or using resonant measurement methods to achieve greater accuracy levels at certain frequencies in terms of loss tangent are promising to gain knowledge for future in situ processing of the lunar regolith [11].

Another possible study could be to increase the measurement range up to 110 GHz, since the Computational and Applied Electromagnetism Laboratory (CAEM-Lab) has a measurement bench for the free space technique, capable of measuring in the V and W bands (from 50 GHz to 110 GHz). Additionally, a study of thermal vacuum at different temperatures in the TVAR environmental chambers at INTA can be performed.

The study on the density variation could also be extended according to the range of frequencies measured; in this way, it could be interesting to see if at higher frequencies, the relative density variations in the powders continue to be linear, as observed in the measurements made between 400 MHz and 2.5 GHz (Figure 7 and Figure 8).

Finally, in the case discussed in Section 4.1 about dried and undried simulants and water content in the simulant, it would be interesting to obtain measurements for water percentages greater than the measurements made, since water on the moon is an important resource. For the search for this resource on the moon, a microwave synthetic aperture radar (SAR) imaging technique could be used [15], and therefore, obtaining the electromagnetic characterization of the material (containing water) seems to be essential.

## Figures and Tables

**Table 1:** Equations for trend lines generated from a linear approximation for the EAC-1A simulant.

Frequency (GHz)	EAC-1A
(0–6] EpsiMu	$\varepsilon^{\prime }$ = −0.0432f + 4.0397 tan $\delta_{e}$ = −0.0015f + 0.0659
[6–20] DAK-3.5	$\varepsilon^{\prime }$ = −0.0432f + 4.0397 tan $\delta_{e}$ = −0.0039f + 0.1429

**Table 2:** Equations for trend lines generated from a linear approximation for the JSC-2A simulant.

Frequency (GHz)	JSC-2A
(0–6] EpsiMu	$\varepsilon^{\prime }$ = −0.0172f + 3.8299 tan $\delta_{e}$ = 0.0005f + 0.0364
[6–20] DAK-3.5	$\varepsilon^{\prime }$ = 0.0136f + 3.4003 tan $\delta_{e}$ = −0.0058f + 0.1550

**Table 3:** List of lunar samples used for the first comparison.

Type of Sample	Acronym	Type	Place
Lunar simulants	JLU	1	Jilin University
	JSC	1	John Space Center
		1A	
		1A (1% CIP)	
		1A (AGGL)	
Lunar regolith	70,051	20	Apollo mission 17

**Table 4:** List of lunar samples used for the second comparison.

Type of Sample	Acronym	Type	Place
Lunar simulants	LHS	1	Lunar highlands simulant
	LMS	1	Lunar mare simulant
	CAS	1	Chinese Academy Science
Lunar regolith	14,163	164	Apollo mission 14
	CE	5	Cheng’E mission

## Data Availability

Data are contained within the article.

## Abbreviations

The following abbreviations are used in this manuscript:

ISRU	In situ resource utilization
OECP	Open-Ended Coaxial Probe
ISR	In situ resource
ESA	European Space Agency
JSC	Johnson Space Center
DAK	Dielectric Assessment Kit
EuCAP	European Conference on Antennas and Propagation
CLEP	China Lunar Exploration Program
CAEM-Lab	Computational and Applied Electromagnetism Laboratory
SAR	Synthetic aperture radar
